# Recruiting to surgical trials in the emergency setting: using a mixed methods study to understand clinician and patient perspectives

**DOI:** 10.1093/bjsopen/zrac137

**Published:** 2022-11-23

**Authors:** Maureen Twiddy, Jacqueline Birtwistle, Amanda Edmondson, Julie Croft, Kathryn Gordon, David Meads, Dermot Burke, Ben Griffiths, Azmina Rose, Peter Sagar, Deborah Stocken, Julia M B Brown, Deena Harji

**Affiliations:** Hull York Medical School, Institute of Clinical and Applied Health Research, Faculty of Health Sciences, University of Hull, Hull, UK; Department of Palliative Care, Leeds Institute of Health Sciences, University of Leeds, Leeds, UK; Department of Psychology, Nottingham Trent University, Nottingham, UK; Leeds Clinical Trials Research Unit, University of Leeds, Leeds, UK; Leeds Clinical Trials Research Unit, University of Leeds, Leeds, UK; Department of Palliative Care, Leeds Institute of Health Sciences, University of Leeds, Leeds, UK; Department of Colorectal Surgery, St James’s University Hospital, Leeds Teaching Hospitals NHS Trust, Leeds, UK; Department of Colorectal Surgery, Manchester University NHS Foundation Trust, Manchester, UK; Patient and Public Involvement Representative for the LaCeS Trial; Department of Colorectal Surgery, St James’s University Hospital, Leeds Teaching Hospitals NHS Trust, Leeds, UK; Leeds Clinical Trials Research Unit, University of Leeds, Leeds, UK; Leeds Clinical Trials Research Unit, University of Leeds, Leeds, UK; Leeds Clinical Trials Research Unit, University of Leeds, Leeds, UK; Department of Colorectal Surgery, Manchester University NHS Foundation Trust, Manchester, UK

## Abstract

**Background:**

Undertaking randomized clinical trials (RCTs) in emergency surgical settings is associated with methodological and practical challenges. This study explored patients’ and clinicians’ perspectives associated with the conduct of an RCT comparing laparoscopic and open colorectal surgery in the acute setting.

**Methods:**

All eligible patients screened and enrolled for the ‘Laparoscopic *versus* open colorectal surgery in the acute setting (LaCeS)’ multicentre, randomized clinical feasibility trial in five UK NHS Trusts were invited to respond to a survey. Patients and healthcare professionals were also invited to take part in semi-structured interviews. Survey and interviews explored the acceptability of the feasibility trial. Interviews were audio recorded, transcribed verbatim, and analysed using thematic analysis. Survey data were analysed descriptively to assess patient views of the trial and intervention.

**Results:**

Out of 72 patients enrolled for the LaCeS RCT, survey data were collected from 28 patients (38.9 per cent), and interviews were conducted with 16 patients and 14 healthcare professionals. Thirteen out of 28 patients (46 per cent) had treatment preferences but these were not strong enough to deter participation. Twelve of the patients interviewed believed that their surgeon preferred laparoscopic surgery, but this did not deter them from participating in the trial. Half of the surgeons interviewed expressed the view that laparoscopic surgery was of benefit in this setting, but recognized that the need for research evidence outweighed their personal treatment preferences. Eight of the 14 recruiters reported that the emergency setting affected recruitment, especially in centres with fewer recruiting surgeons. Interviewees reported that recruitment was helped significantly by using surgical trainees to consent patients.

**Conclusion:**

This study identified specific challenges for the LaCeS trial design to address and adds significant insights to our understanding of recruiting to emergency surgical trials more broadly.

## Teaser

This paper presents the findings of a mixed-methods study exploring patient and clinician views of participating in the LaCeS trial, comparing laparoscopic and open colorectal surgery in the acute setting. Patients found the trial and intervention acceptable, but the emergency setting affected recruitment.

## Introduction

Recruitment to surgical randomized clinical trials (RCTs) continues to be challenging^1–3^, with 20 per cent discontinued early, most commonly due to recruitment difficulties^3^. Qualitative research provides an opportunity to identify recruitment challenges by exploring patients’ and healthcare professionals’ experiences^4^.

When recruiters are not in a state of personal clinical equipoise, they can be reluctant to approach patients who they feel are better suited to one treatment than the other. Recruitment efforts can also be undermined by several issues, including the difficulties communicating the trial aims, problems describing the concept of randomization, and the concern about the effect of recruitment on the doctor–patient relationship. There is also the added workload involved with a trial to be considered^5,6^.

Patient-level recruitment barriers are complex and often trial specific^7^, and include difficulties understanding the concept of randomization, and patients wanting their clinician to choose the ‘best’ treatment for them^8,9^. Clinical trials in acute medicine investigate seriously ill or injured patients, which makes it more difficult for them to reach an informed decision about trial involvement^10^.

Emergency general surgery is one of the most common reasons for admission to hospital. Of the 30 000 people a year undergoing major abdominal surgery, a third are for emergency colorectal pathology^11^.

The aim of this study was to investigate patient and clinician perceptions of the intervention (laparoscopic surgery) and trial processes to improve trial recruitment.

## Method

The ‘Laparoscopic *versus* open colorectal surgery in the acute setting’ (LaCeS) multicentre randomized feasibility trial^12^ recruited from five UK NHS Trusts and randomized patients presenting with acute colorectal pathology requiring resection, to receive laparoscopic or open surgery. The trial was registered with registration number: ISRCTN15681041 (http://www.controlled-trials.com). The target sample size was 66 patients, and 64 patients were randomized to the trial^12^. Two previous emergency colorectal surgery trials closed early, in part due to poor recruitment^13,14^. LaCeS was conducted between July 2016 and November 2017 and included qualitative work to understand patient and recruiter experiences to identify recruitment challenges to inform the development of a training package for a definitive trial. Interviews were undertaken by researchers who were independent of the trial recruitment team. Patient and staff interviews were audio recorded (notes were taken for one staff interview), then transcribed verbatim for analysis and anonymized. *Supplementary material* reports an overview of the thematic structure.

UK NHS Research Ethics Committee (REC) approval was granted for the study (REC reference: 15/HY/0542).

### Design

The data collection and analysis were underpinned by a pragmatic interpretative framework^15^, with both quantitative (survey) and qualitative (interview) data collected to identify key barriers and levers to trial recruitment. The mixed-methods approach was used to gather the feedback from as many eligible patients as possible, accepting that many would not consent for interview. The survey provided an overview of patient views of the trial and informed the sampling for the interviews, including the identification of topics to explore at interview. The interviews provided the opportunity to explore issues identified by the survey in depth; both survey and interviews were given equal status. Interviews with staff explored the challenges to recruiting in the emergency setting and identified ways that we could support patient recruitment.

### Patient survey and interviews

All patients approached for trial involvement were invited to complete a short (paper copy) survey about the recruitment and randomization process 7 days after surgery. Patients completed the questionnaires independently, and responses were anonymous. Questions asked patients their views about being approached for the trial and the intervention options, with participants asked to respond on a 1 to 5 Likert scale, with strongly disagree, indicated by 1 and strongly agree indicated by 5 (*Supplementary material*). To encourage participation no demographic information was collected.

All patients approached for the trial were also eligible to be interviewed. Patients were invited to participate in a semi-structured face-to-face (own home) or telephone interview to discuss their experiences of trial recruitment and participation (*Supplementary material*). Interviews continued until no new concepts were found during analysis^16^. Interviews were conducted by one researcher between September 2016 and October 2017.

### Staff interviews

E-mail invitations were sent to 20 staff from the five sites (three to five staff per site), to ensure that staff of all levels on the recruitment log were represented (consultants, surgical trainees, nurses, and research nurses), including surgeons on the recruitment log who had not approached patients for the trial. Two researchers conducted telephone or face-to-face (in hospital) interviews. Separate topic guides were developed for patients and staff using the existing literature^2,17,18^ (*Supplementary material*).

### Outcomes of interest

The outcome of interest was to evaluate patient views of the surgery and experiences of being approached for and taking part in the trial; also, the staff experiences running the trial, and approaching and consenting patients to the trial were explored.

### Data analysis

Survey data were analysed using descriptive statistics. The qualitative sample size was not pre-determined for this analysis, but purposively selected to ensure diversity in age, intervention received, and sex. Line-by-line coding used an inductive approach, with a focus on content and meaning. Codes were grouped and a matrix was used to structure the data using the principles of thematic analysis^19^ in Nvivo^20^. Patient and staff interviews were analysed separately. In each case, the first four transcripts were independently coded using an inductive approach by two experienced, postgraduate qualitative researchers. Codes were compared and a coding framework was agreed. The coding framework was then applied to the remaining transcripts. Codes were discussed and recategorized and themes were generated by authors with regular checking of the data. The data quality from phone and face-to-face interviews was assessed by examining the length of time that topics were discussed for each format; phone interviews were marginally shorter, but all topics were covered. The survey data and interview findings were then cross-tabulated to identify where the qualitative data provided explanation for the survey results, and these are presented within the analysis. The analysis was further refined by discussion of the initial themes and subthemes in trial meetings that included clinical colleagues, and the patients’ representative involved in the study. Patients’ perspectives were analysed, and the following topics were identified: laparoscopy *versus* open surgery, randomization, and clinical equipoise. Clinicians’ perspectives were analysed for the commitment to the trial, patients’ exclusion, their response to patients’ preferences, and the impact of the on-call rota on recruitment.

## Results

At the close of trial recruitment, 28 of 72 (38.9 per cent) patients approached for the trial had completed feedback questionnaires (27 consenters and one decliner)^12^.

Twenty-two patients (30.5 per cent) approached for the trial consented to an interview and 16 trial participants (22.2 per cent) were interviewed. In total, only eight people declined trial participation^12^, and no trial decliners consented to interview (*Supplementary material*). Interviewees reflected the balance between trial arms, with interviews conducted with 6 out of the 33 patients randomized to laparoscopic surgery, and with 7 out of 31 randomized to open surgery. Younger trial participants were more likely to consent to interview, with 5 out of the 16 people aged 18–49 years (31.2 per cent) who took part in the trial consenting to interview, compared with only 1 patient out of the 12 (8.3 per cent) who were aged more than 80 years. Patient interviews lasted 36 to 80 min, and no new information was gained after 13 interviews, but continued for a further three interviews to ensure no new ideas were missed. Fourteen of the 20 staff approached (70 per cent) consented to interview, all of whom were actively involved in trial recruitment.

Staff interviews lasted between 22 and 72 min and no new ideas were identified after 12 interviews so were stopped at 14 participants (*Supplementary material*).

Quotes are provided to support the analysis. Sex, age range, and procedure are provided for patients. The role of the clinician is provided alongside quotes to aid interpretation.

### Patient perspectives

Interviewees were aged from 18 years to 80 years, with all age ranges represented (*Supplementary material*). All respondents were of white ethnicity, and all participated in the trial. Presenting problems included diverticular disease and inflammatory bowel disease (further details are not provided as this may identify individuals). Five patients opted for telephone interviews; the remainder were interviewed face to face in the patient’s home or on university premises. Thirteen out of 28 patients (46 per cent) patients had treatment preferences but these were not strong enough to deter participation.

**Fig. 1:**
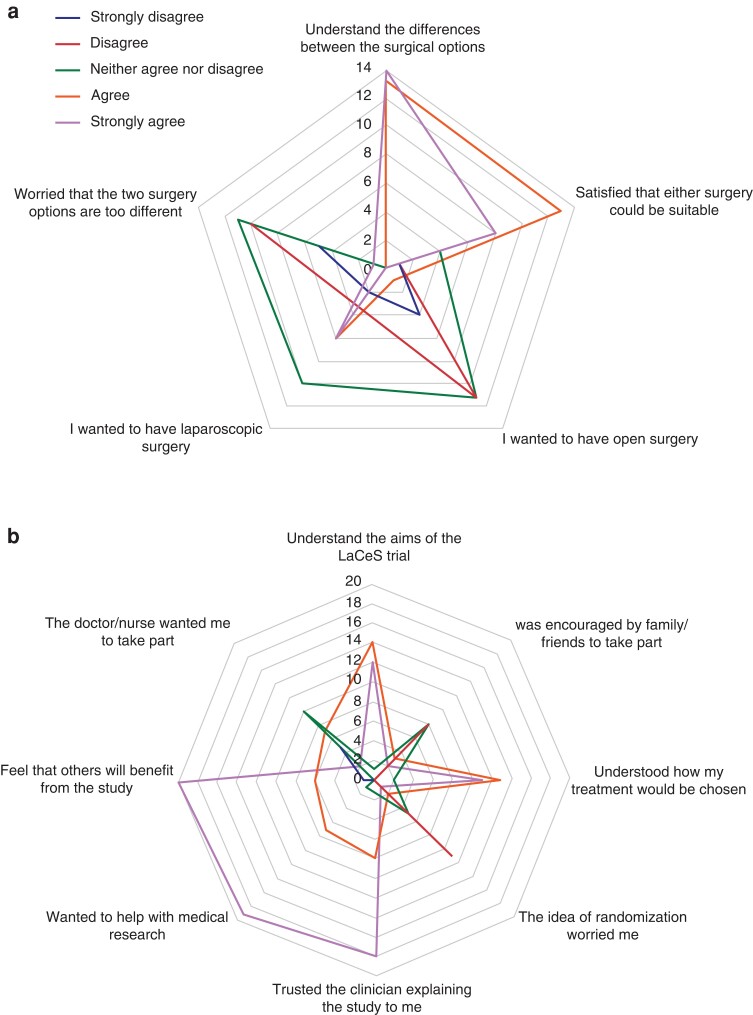
Radar plots **a** Patient views of the surgery options. **b** Patient views of the trial (survey responses). .

**Fig. 2:**
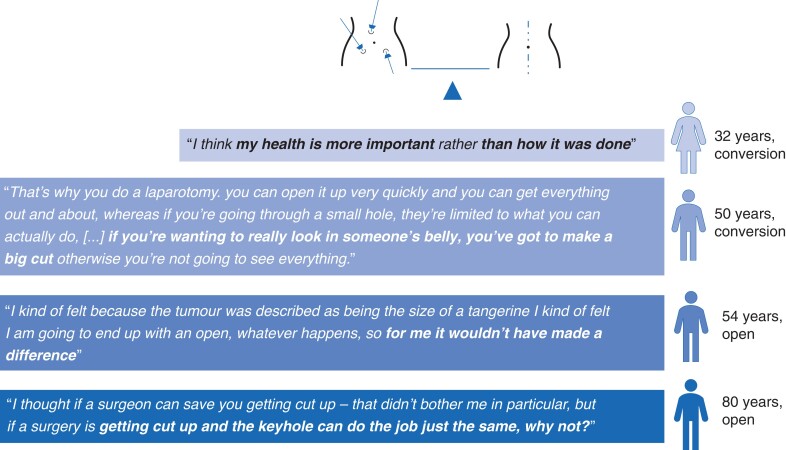
Balancing laparoscopy *versus* open surgery: patients’ perspectives

The survey investigated whether the patients understood the aims of the trial and the difference between the treatment options. Most consenting patients believed that both surgical options were appropriate for them (on the Likert scale, the median for consenters was 4), although there was a slight treatment preference towards laparoscopic surgery (median for consenters of 3), compared with preference for open surgery (median of 2) (*Fig. 1*). Some interviewees reported that their previous experience of surgery (both open and laparoscopic) influenced their decision to take part in the trial, as the trial gave them a chance of receiving laparoscopic surgery. However, others focused on the importance of a good outcome, rather than the mode of access. Despite a general preference for laparoscopic surgery, more than half of interviewees believed that open surgery would facilitate better visualization of the surgical field and ensure that things would not be ‘missed’ but recognized the cost of open surgery was significant scarring and an increased risk of infection and hernia. A few agreed to be randomized even though they were convinced that they would need open surgery anyway. Most patients believed that laparoscopic surgery would shorten their hospital stay but valued the reassurance that their operation would be converted to open, if necessary. *Fig. 2* provides quotes that demonstrate patient perspectives related to this topic.

The survey indicated that patients were generally not worried about randomization (on the Likert scale, median of 5), and most agreed (25 patients, 89 per cent) with the statement ‘I understood how my treatment option would be chosen’ (median of 4) (*Supplementary material*); however, the interviews revealed that a small minority misunderstood what randomization actually involved. Survey data showed that participants trusted their doctor’s explanation of the study (median of 5), and that they participated to help with medical research (median of 5), or so that other patients would benefit in the future (median of 4), a view that was echoed in the interviews. Patients were largely in personal equipoise, feeling that given the evidence, both options were appropriate for them. Patients expanded on their reasons in the interviews, with trust in their clinical team being a key driver to accepting the trial. Patients who were very unwell, or processing news of a serious diagnosis recalled little about being approached for the trial but remembered the reassurance that the clinical team provided. For a small minority, a family member helped them make the decision to participate, and none regretted involvement.

**Fig. 3:**
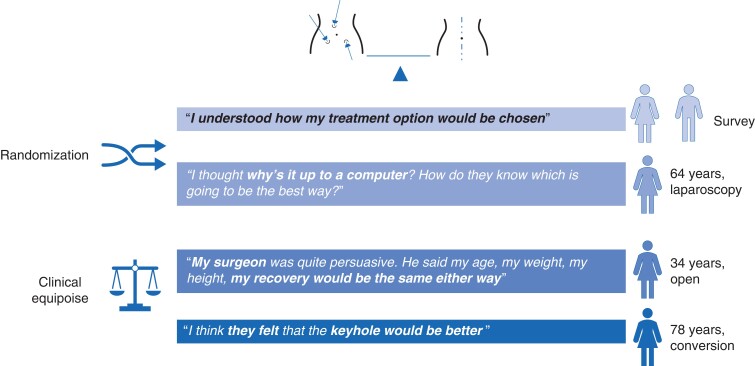
Randomization and clinician equipoise: patients’ perspectives

The survey data suggested that on the whole patients did not feel that their surgeon had a particular preference for one treatment over the other, but some interviewees also noted that the surgeon was taking part in the trial and so they assumed that their surgeon felt the question needed answering, or that they were in favour of laparoscopic surgery. Patients did not think there was anything unusual about this and were not concerned. *Figure 3* provides example quotes regarding patient views about equipoise.

### Clinician perspectives

**Fig. 4:**
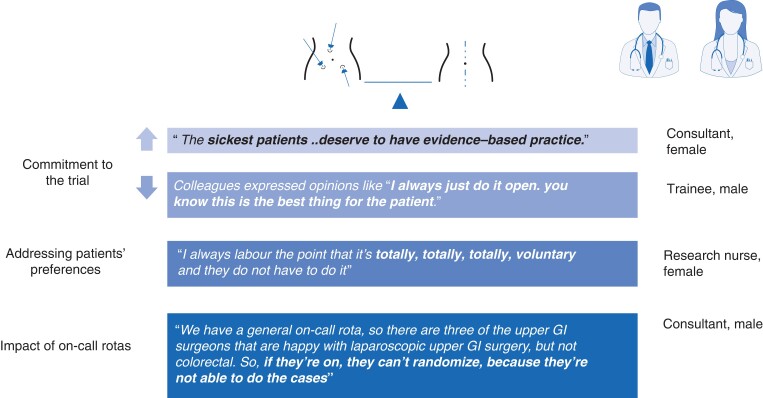
Clinicians’ perspectives on the randomized clinical study GI, gastrointestinal.

Half of the surgeons interviewed expressed the view that laparoscopic surgery was of benefit in this setting, and nine (64 per cent) believed that the need for research evidence outweighed their personal treatment preferences (*Fig. 4*).

Interviewees provided possible reasons for colleagues not participating in the trial, including: a lack of awareness of the trial, lack of personal clinical equipoise, and a belief that the trial will be difficult to recruit to. Surgeons occasionally expressed initial regret about offering the trial to a patient and were surprised by the positive outcome (*Fig. 4*).

Despite their claim that they could set aside their own personal views, four out of eight consultants (50.0 per cent) expressed unwillingness to enter patients with ulcerative colitis into the trial as there was a strong belief that the benefits of reduced scarring from laparoscopic surgery outweighed any possibility of poorer outcomes, and so clinicians often did not raise the idea of the trial to these patients.

Recruiters recalled that few patients expressed a treatment preference, but when they did, recruiters were often reluctant to discuss these with the patient; some citing a belief that this was not ethical, others believing that if the patient provided a sensible justification, that this should be accepted (*Fig. 4*).

Finally, at most sites, specialist trainees were involved in identifying and recruiting patients but the unpredictability of cases, together with the on-call rota meant that some eligible patients could not be approached because the surgeon on call could not operate laparoscopically on patients requiring lower gastrointestinal surgery. In other instances, identified patients could not be operated on because the on-call surgeon was not in personal equipoise and refused to enter the patient into the trial. When only one or two surgeons were involved in the trial, the on-call rota meant that recruitment patterns were sporadic, with significant intervals where no patients could be approached, making monthly recruitment rate targets inappropriate. Surgeons at these sites were frustrated about how many eligible patients were missed (*Fig. 4*).

## Discussion

The aim of the LaCeS feasibility trial was to establish whether it is possible to recruit to a surgical trial in the emergency setting and to ensure that trial processes and the intervention were acceptable to patients and healthcare professionals^12^. The trial was acceptable to patients but some selection bias on the part of the recruiters, a lack of engagement by some clinicians, and a lack of personal clinical equipoise on the part of some clinicians were barriers to recruitment.

In this trial, potentially eligible patients were identified by specialist trainees and consultant surgeons. This approach worked well, but the complex on-call rotas meant that some patients were not recruited because there was no participating surgeon on call. This is in sharp contrast to the elective setting where the flow of patients is steady and relatively predictable. In common with other trials, recruitment practices could be at odds with the trial protocol^12^. Staff interviews revealed that when recruiting patients with ulcerative colitis, clinicians were reluctant to risk patients receiving open surgery. This meant that some patients were actively excluded from the trial by clinicians, a problem that has been reported elsewhere^3^. The need for personal equipoise is debated^21^, but staff interviewees suggested that some surgeons did not recruit due to an absence of personal equipoise, and clinician preference has been identified in a recent survey as a key reason that trials fail to recruit^3^.

The survey showed that patients were not worried about randomization, but the interviews revealed some misconceptions; in particular around the nature of randomization. This phenomenon is well recognized^22^. In the emergency setting time to discuss the trial is limited and patients may be anxious, and this may contribute to this difficulty; however, as recruitment interviews were not recorded we cannot determine where the issue lies.

Clinicians believed that patients would have a strong preference for laparoscopic over open surgery, but the survey and patient interview data, as well as the recruitment data^12^, do not support this conclusion. The interviews provide insights into the reasons for this. Unlike studies with a placebo arm, which are often difficult to recruit to, all patients were to receive their planned surgery and only the mode of access to the abdomen varied. For most patients, the outcome of their surgery was viewed as more important than how surgical access was achieved. Although laparoscopic surgery was viewed as preferable to open surgery because of the reduced risk of infections and quicker healing, patients also believed a keyhole approach reduced the visual field, and some worried that things might be missed, and were reassured that their surgeon could revert to an open procedure if needed.

The findings reported are helpful for understanding some of the specific recruitment issues around recruiting to trials in the emergency setting. Only eight eligible patients declined trial participation^12^, which speaks strongly to the nature of the interventions compared in this trial. In contrast to many surgical trials, both interventions were surgical, and so were viewed as comparable (and generally acceptable) by patients. Although patients participating in the trial were interviewed, no trial decliners consented to an interview, so the voices of this group are missing from the data. The response rate to the patient feedback survey was also low (38.9 per cent). Recruiters were invited to offer the survey to all eligible patients, but staff were reluctant to ask patients to complete the survey when they had declined the trial. The staff taking part in the trial were also interviewed, and their experiences was collected, but clinical staff who were on the recruitment log, but did not recruit any patients, declined participation, so this study offers only a partial understanding of the reasons why some staff did not engage well with the trial.

This paper adds to the literature on patient recruitment in the emergency setting. The recruitment was helped by the support of junior doctors identifying eligible patients and hindered by on-call rotas, clinical pathways, and a lack of personal clinical equipoise. Monthly recruitment targets were unsuitable for the sporadic nature of emergency surgery and for trials in the emergency setting and so 3-monthly targets may be more helpful; unlike elective surgery, emergency surgery is responsive, so recruitment rates cannot be managed in the same way.

## Supplementary Material

zrac137_Supplementary_Data

## Data Availability

Data are not available as permission was not requested from participants at the time of study.

## References

[1] McDonald AM , Knight RC, Campbell MK, Entwistle VA, Grant AM, Cook JA et al What influences recruitment to randomised controlled trials? A review of trials funded by two UK funding agencies. Trials 2006;7:9 16603070 10.1186/1745-6215-7-9 PMC1475627

[2] Donovan JL , Paramasivan S, de Salis I, Toerien M. Clear obstacles and hidden challenges: understanding recruiter perspectives in six pragmatic randomised controlled trials. Trials 2014;15:5 24393291 10.1186/1745-6215-15-5 PMC3892115

[3] Crocker J , Farrar N, Cook J, Treweek S, Woolfall K, Chant A et al Recruitment and retention of participants in UK surgical trials: survey of key issues reported by trial staff. BJS Open 2020;4:1238–1245 33016008 10.1002/bjs5.50345 PMC7709375

[4] Elliott D , Husbands S, Hamdy S, Holmberg L, Donovan J. Understanding and improving recruitment to randomised controlled trials: qualitative research approaches. Eur Urol 2017;72:789–798 28578829 10.1016/j.eururo.2017.04.036

[5] Donovan J , de Salis I, Toerien M, Paramasivan S, Hamdy F, Blazeby J. The intellectual challenges and emotional consequences of equipoise contributed to the fragility of recruitment in six randomised controlled trials. J Clin Epidemiol 2014;67:912–920 24811157 10.1016/j.jclinepi.2014.03.010 PMC4067744

[6] Fletcher B , Gheorghe A, Moore D, Wilson S, Damery S. Improving the recruitment activity of clinicians in randomised controlled trials: a systematic review. BMJ Open 2012;2:e000496 10.1136/bmjopen-2011-000496 PMC3253423 22228729

[7] O’athain A , Thomas K, Drabble S, Rudolph A, Hewison J. What can qualitative research do for randomised controlled trials? A systematic mapping review. BMJ Open 2013;3:e002889 10.1136/bmjopen-2013-002889 PMC3669723 23794542

[8] Featherstone K , Donovan JL. “Why don’t they just tell me straight, why allocate it?” The struggle to make sense of participating in a randomised controlled trial. Soc Sci Med 2002;55:709–719 12190265 10.1016/s0277-9536(01)00197-6

[9] Whybrow P , Pickard R, Hrisos S, Rapley T. Equipoise across the patient population: optimising recruitment to a randomised controlled trial. Trials 2017;18:140 28347354 10.1186/s13063-016-1711-8 PMC5369002

[10] Rowlands C , Rooshenas L, Fairhurst K, Rees J, Gamble C, Blazeby J. Detailed systematic analysis of recruitment strategies in randomised controlled trials in patients with an unscheduled admission to hospital. BMJ Open. 2018;8:e018581 10.1136/bmjopen-2017-018581 PMC5829602 29420230

[11] NELA Project Team . Seventh Patient Report of the National Emergency Laparotomy Audit. London: RCoA, 2021

[12] Harji DP , Marshall H, Gordon K, Twiddy M, Pullan A, Meads D et al Laparoscopic versus open colorectal surgery in the acute setting (LaCeS trial): a multicentre randomized feasibility trial. Br J Surg. 2020;107:1595–1604 32573782 10.1002/bjs.11703

[13] Oberkofler CE , Rickenbacher A, Raptis DA, Lehmann K, Villiger P, Buchli C et al A multicenter randomized clinical trial of primary anastomosis or Hartmann’s procedure for perforated left colonic diverticulitis with purulent or fecal peritonitis. Ann Surg 2012;256:819–826; discussion 26–27 23095627 10.1097/SLA.0b013e31827324ba

[14] Binda GA , Karas JR, Serventi A, Sokmen S, Amato A, Hydo L et al Primary anastomosis vs nonrestorative resection for perforated diverticulitis with peritonitis: a prematurely terminated randomized controlled trial. Colorectal Dis 2012;14:1403–1410 22672447 10.1111/j.1463-1318.2012.03117.x

[15] Patton M . Qualitative evaluation and research methods. Newbury, CA: Sage, 1990

[16] Malterud K , Siersma V, Guassora A. Sample size in qualitative interview studies: guided by information power. Qual Health Res 2016;26:1753–1760 26613970 10.1177/1049732315617444

[17] Blazeby J . Recruiting patients into randomized clinical trials in surgery. Br J Surg 2012;99:307–308 22237652 10.1002/bjs.7818

[18] Elliott D , Husbands S, Hamdy FC, Holmberg L, Donovan JL. Understanding and improving recruitment to randomised controlled trials: qualitative research approaches. Eur Urol 2017;72:789–798 28578829 10.1016/j.eururo.2017.04.036

[19] Braun V , Clarke V. Reflecting on reflexive thematic analysis. Qual Res Sport Exerc Health 2019;11:589–597

[20] QSR International . NVivo (released March 2020). 2020

[21] Weijer C , Shapiro SH, Glass KC. For and against: clinical equipoise and not the uncertainty principle is the moral underpinning of the randomized clinical trial. BMJ 2000;321:756–758 10999914 10.1136/bmj.321.7263.756 PMC1127868

[22] Wade J , Donovan J, Lane J, Neal D, Hamdy F. It’s not just what you say, it’s also how you say it: opening the ‘black box’ of informed consent appointments in randomised controlled trials. Soc Sci Med 2009;68:2018–2028 19364625 10.1016/j.socscimed.2009.02.023

