# Supplementary material for: Recruiting to surgical trials in the emergency setting: using a mixed methods study to understand clinician and patient perspectives
Source: BJS Open. 2022 Nov 23;6(6):zrac137. doi: 10.1093/bjsopen/zrac137 (PMC9683391; doi:10.1093/bjsopen/zrac137)
Supplement: zrac137_Supplementary_Data [file zrac137_supplementary_data.docx]

# Recruiting in surgical trial in the emergency setting: understanding clinician and patient perspectives: mixed methods study

Authors

Maureen Twiddy 1, Jacqueline Birtwistle2, Amanda Edmondson 3, Julie Croft4, Kathryn Gordon 4, David Meads2, Dermot Burke5, Ben Griffiths6, Azmina Rose7, Peter Sagar5, Deborah Stocken4, Julia MB Brown4 and Deena Harji 4,6 for an on behalf of the LaCeS study team

1. Hull York Medical School, Institute of Clinical and Applied Health Research, University of Hull, Hull

2. Leeds Institute of Health Sciences, University of Leeds, Leeds

3. Department of Psychology, Nottingham Trent University

4. Leeds Clinical Trials Research Unit, University of Leeds, Leeds

5. Department of Colorectal Surgery, St James’s University Hospital, Leeds Teaching Hospitals NHS Trust, Leeds

6. Department of Colorectal Surgery, Manchester University NHS Foundation Trust, Manchester

7. Patient and Public Involvement Representative for the LaCeS Trial

**Corresponding author.** Dr Maureen Twiddy, Hull York Medical School, Institute of Clinical and Applied Health Research, University of Hull, Cottingham Road, Hull, HU6 7RX, UK (e-mail: maureen.twiddy@hyms.ac.uk)

**ORCID ID: 0000-0002-3794-1598**

**Supplementary Materials - Index**

| **Supplementary Methods** |  |
| --- | --- |
| Patient Survey | *pag. 2* |
| Topic guides (patient and staff) | *pag. 5* |
| **Supplementary Results** |  |
| Demographic data (patients and staff) | *pag. 11* |
| Survey Responses – descriptive statistics | *pag. 13* |
|  |  |

# Supplementary Methods

**LaCeS Patient Feedback Questionnaire**

You have been approached to see if you would be interested in taking part in a study looking at open versus laparoscopic surgery for patients requiring emergency bowel surgery (the LaCeS trial). This is a feasibility trial and the main aim is to find out if people are willing to take part in trials in this setting, therefore the reasons why you decided to take part or to not take part will help us to find out if we could run a larger trial in the future to compare the two surgical options. You may have agreed to join the study OR you may have decided not to take part. Either way, we would value your feedback, however, this questionnaire is optional and you do not have to complete it if you do not want to.

If you have decided not to take part, we fully respect that decision and your feedback is valuable to us regardless of the choice you made. We are NOT asking you again to take part in the study.

The questionnaire is completed anonymously and neither your doctor nor nurse will see your answers if you do not want them to. Once you have completed the questions below please place the questionnaire in the attached envelope. This can then be posted directly to the research office who are conducting the study (the envelope is freepost), or handed back to the staff in clinic who will post it on your behalf.

**Please answer a few questions about yourself. Circle the right answer or fill in the space provided.**

1. Who explained most of the LaCeS feasibility trial detail to you? Doctor/nurse/other
2. Are you taking part in the LaCeS feasibility trial? Yes/No
3. If you are taking part, did you provide consent yourself prior to surgery, or were you entered into the trial by a relative who agreed on your behalf?

   Yes, I consented myself/ No a relative advised on my behalf
4. Please circle the number that represents how you feel about the following statements regarding your decision to take part or not in the LaCeS feasibility trial.
    1= strongly disagree, 4=disagree, 3= neither agree nor disagree 4= agree 5=strongly agree

|  | SD | D | Neither | A | SA |
| --- | --- | --- | --- | --- | --- |
| I understand the aims of the LaCeS feasibility trial | 1 | 2 | 3 | 4 | 5 |
| I understand the differences between the surgical options involved | 1 | 2 | 3 | 4 | 5 |
| I was satisfied that either surgery could be suitable for me | 1 | 2 | 3 | 4 | 5 |
| I wanted to have open surgery | 1 | 2 | 3 | 4 | 5 |
| I wanted to have laparoscopic surgery | 1 | 2 | 3 | 4 | 5 |
| I was encouraged by some family/friends to take part | 1 | 2 | 3 | 4 | 5 |
| I understood how my treatment (open or laparoscopic) would be chosen (Randomisation) | 1 | 2 | 3 | 4 | 5 |
| The idea of randomisation worried me | 1 | 2 | 3 | 4 | 5 |
| I trusted the doctor/nurse explaining the study to me | 1 | 2 | 3 | 4 | 5 |
| I wanted to help with the medical research | 1 | 2 | 3 | 4 | 5 |
| I feel that others with my illness will benefit from the study results | 1 | 2 | 3 | 4 | 5 |
| I am worried that the two surgery options in the trial are too different | 1 | 2 | 3 | 4 | 5 |

**Did you read the participant information sheet? Yes/No**

**If yes, was it helpful? Yes/No**

**Was the amount of information in it too little? Just right? Too much?**

**Have you any comments on the participant information sheet? ___________________________

__________________________________________________________________________________**

**If you have any other reasons for deciding to take part or not please write below: _____________**

**Did any other information help you make your decision? _________________________________**

**If you are not taking part we would appreciate it if you could tell us the main reason why: _______

__________________________________________________________________________________**

**Do you have any other comments about the trial? ______________________________________**

**Thank you very much for your time.**

**LaCeS Patient Interview topic guide**

**Opening question: Ask the patient to talk about their condition and confirm the treatment they have had for this.**

**Can you describe in your own words what the LACES study is about?** Identify the participants’ actual understanding and misunderstanding about the trial.

**Why did you a) decide to take part/decline the LaCeS study?**

**Did you already have a specific treatment preference in mind before the study was presented to you**? What impact did the study information have on this?

**What did you think about the fact that the treatments are randomised**?

**What do you think of the risks of taking part in this study?**

For those entered under consultee advice: (in italics)

*You were entered into the trial by a family member, what did you feel about this process?*

*Did you decide to continue with the trial once it was explained to you?*

*Do you know how your family member felt about being asked to make this decision for you?*

*Is asking a family member to make this decision appropriate?*

*For those who did not continue with the tria*l

**Decliners only** *Can you tell me why you decided not to take part?*

**Who explained the trial to you**? Do you believe they covered all the things that needed to be discussed?

**What written information did you receive?** (explore understanding and acceptability of this). How could it be improved? E.g. format - audio or film?

**Did you talk to anyone about taking part in the study**? friends, family etc.? If yes, what advice was given about taking part?

For those entered under consultee advice:

*Did your family tell you about trial? How did they explain it to you?*

*Who else would you have liked to talk to about the decision to enter you into the trial?*

**What do you think are the disadvantages and advantages of this study**? (explore how the participants weigh up pros and cons)

**For Non-consenters:**

*What were your main concerns about taking part in this study? Prompts: lack of interest/time/ other things of greater concern at this time?*

*In your experience of being offered this trial, was there anything we could have changed so you might have considered participating?*

**When we design a study like this we have to decide what will determine the ‘success’ of the intervention (laparoscopic treatment). What is a good outcome for patients? What is important to you**? (examples to offer reduced length of stay, improved quality of life, reduced pain, reduced physiological burden, and reduced time to recovery).

**Some patients come back to the hospital to complete the follow-up questionnaires. How have you completed your follow up questionnaires**? What would you feel about completing these by phone/post? Did you need help needed to complete these?

**Only for those who had surgery - Blinding**

**Were you happy not to know what procedure you had**? Did you guess which group you were in prior to being told. Were you correct in your guess? What made you reach this conclusion?

If we were to ask patients to wear an eye mask until the patient is unblinded at 7 days while the nurse checks the wound – how would you feel about this?

Were you left with any concerns by not knowing what treatment you had?

What could we do better?

**Only for non-consenters**

**Did the fact that you would not know which procedure you had influence your decision not to participant in the trial**?

Do you have anything else to add?

**Staff Interview Topic Guide - LaCeS Feasibility**

Background information about the participant - role in the project, role in the recruitment and consent process

**Can you describe in your own words what the LaCeS trial is about**? (Prompts: Identify the participants understanding and any misunderstanding about the trial).

**What was your experience of presenting the trial to patients or in supporting patients who were part of the trial?**

Prompts: In your experience were patients happy to be part of the trial? Did patients have specific treatment preferences prior to the discussion? How were these explored? Did you encounter any patients that had misconceptions about this trial or research in general?

**How easy or difficult has recruitment to this trial been**?

Are there any issues with the eligibility criteria that have made recruitment difficult? Do you think all eligible patients have been approached? Do you feel you are excluding any patients that you feel should be included? If so, who and why?

**If you do not think all eligible patients have been approached, why might this be? What can we do to improve this**?

**What is your opinion of the written resources that were provided to the patient?**

Prompts: Was the patient information sheet adequate? Could it be improved in any way?

**Can you tell me about your experience running the trial?**

**How did you find the process of randomisation?**

**Were you able to access the resources you needed to run the trial effectively**? (e.g. theatre access, laparoscopic kit) Were these easy to access? If not, what problems did they cause for the running of the trial?

**Were there any difficulties recruiting patients? Could you tell me about these?**

**The study collects quite a lot of data on patients. How do you feel about the timing of data collection**? (3/7/30 days – then 3/6/12 month). Do you feel we have the follow up times right?

Do we need to follow up to 12 months?

**What type of support is given to patients who take part in studies in this area (different surgical procedures**)? Do you think people are given enough information and support? Do you have any recommendations or suggestions?

**Do you think it’s appropriate and acceptable to use this type of trial design for emergency surgery**?

What are the key issues? opportunities and challenges? Randomisation? Is there anything you would change?

**What challenges did you face presenting the treatments in a balanced way**? Prompts: What do you think are the disadvantages and advantages for patient taking part in this trial? How did you feel explaining the (medical) uncertainty concerning the relative benefits of these 2 approaches?

How did patients respond when told we don’t know which is best?

**Were you provided with training or resources to support you in recruiting patients to this stud**y? What type of resources/training were offered? Have you had time to use any resources? Previous experience on similar types of trials? How could the resources/training be improved?

**Whilst being involved in this study how have you been able to balance your clinical and research roles?**

(Prompts: Were there any issues that made it difficult? Were you supported to deliver in both roles?

Were there any issues that made it more difficult? Do you have any suggestions or recommendations about how we can improve the design to make things easier for you?)

**One of the important issues is for us to determine what the primary outcome for the main trail should be. Having taken part, what are your thoughts on this**? (Length of stay, quality of life, pain, physiological burden, time to recovery)

**What do you think of the blinding procedures used in the trial?**

Keeping the patient blind to their allocation can be challenging – there has been a suggestion that we give patients an eye mask to wear during the assessment of the wound – what do you feel about this?

**Consultee advice – have you used it?** How easy was it to identify a suitable Consultee?

Did you feel confident about approaching Consultees and entering patients based on Consultee advice? What issues, if any did you identify using the form? Could this process be made simpler?

How do you feel about gaining consent form patients once they have regained capacity?

**Are there any other things we have not discussed about the trial design that you think should be changed when we go into phase III**?

# Supplementary Results

**Table 1: Clinician and patient characteristics (interviews and trial)**

|  | **Number of interview participants (gender)** | **Trial sample**  **Participants**  **(Gender not reported by main trial)** |
| --- | --- | --- |
| **Clinician type** |  |  |
| Consultant surgeon (including local PI at each site) | 8 (7 male: 1 female) | n/a |
| Specialist trainee | 3 (2 male: 1 female) | n/a |
| Research nurse | 3 (0 male: 3 female) | n/a |
| **Patients** |  |  |
| Randomised to Laparoscopic | 6 (3 male: 3 female) | 33 |
| Laparoscopic converted to open during surgery | 3 (1 male: 2 female) | 13* |
| Randomised to Open surgery | 7 (5 male: 2 female) | 31 |
| Trial decliners | 0 | 8 |
| **Age range** |  |  |
| 18-49 | 5 (3 male: 2 female) | 16 |
| 50-59 | 3 (2 male: 1 female) | 8 |
| 60-69 | 5 (3 male: 2 female) | 17 |
| 70-79 | 2 (0 male: 2 female) | 11 |
| >80 | 1 (1 male: 0 female) | 12 |

*conversion to open surgery participants are a subset of those randomised to laparoscopic.

**Descriptive statistics from survey responses**

|  | Trial consenters | | | | Declined participation | |
| --- | --- | --- | --- | --- | --- | --- |
|  | Valid | Missing | Mean | SD | Valid | Mean |
| I understand the aims of the LaCeS feasibility trial | 27 | 0 | 4.41 | .572 | 1 | 5.00 |
| I understand the differences between the surgical options involved | 27 | 0 | 4.56 | .506 | 1 | 4.00 |
| I was satisfied that either surgery could be suitable for me | 27 | 0 | 4.07 | .917 | 1 | 2.00 |
| I wanted to have open surgery | 27 | 0 | 2.22 | .751 | 1 | 4.00 |
| I wanted to have laparoscopic surgery | 27 | 0 | 3.52 | 1.189 | 1 | 2.00 |
| I was encouraged by some family/friends to take part | 27 | 0 | 2.41 | 1.248 | 1 | 3.00 |
| I understood how my treatment (open or laparoscopic) would be chosen (Randomisation) | 26* | 1 | 4.38 | .637 | 1 | 4.00 |
| The idea of randomisation worried me | 27 | 0 | 2.07 | 1.072 | 1 | 3.00 |
| I trusted the doctor/nurse explaining the study to me | 27 | 0 | 4.63 | .565 | 1 | 5.00 |
| I wanted to help with the medical research | 27 | 0 | 4.67 | .555 | 1 | 5.00 |
| I feel that others with my illness will benefit from the study results | 27 | 0 | 4.63 | .839 | 1 | 5.00 |
| The doctor/nurse wanted me to take part | 27 | 0 | 3.00 | 1.271 | 1 | 3.00 |
| I am worried that the two surgery options in the trial are too different | 27 | 0 | 2.26 | .944 | 1 | 3.00 |

Questions scored on a 1-5 scale: strongly disagree=1, disagree = 2, neither agree nor disagree =3, agree=4, strongly agree=5. * one participant failed to answer one question.
